# Semi-supervised Label Generation for 3D Multi-modal MRI Bone Tumor Segmentation

**DOI:** 10.1007/s10278-025-01448-z

**Published:** 2025-02-20

**Authors:** Anna Curto-Vilalta, Benjamin Schlossmacher, Christina Valle, Alexandra Gersing, Jan Neumann, Ruediger von Eisenhart-Rothe, Daniel Rueckert, Florian Hinterwimmer

**Affiliations:** 1 Department of Orthopedics and Sports Orthopedics, Klinikum Rechts Der Isar, Technical University of Munich, Ismaninger Strasse 22, 81675 Munich, Germany; 2 Institute for AI and Informatics in Medicine, Technical University of Munich, Einsteinstrasse 25, 81675 Munich, Germany; 3 Musculoskeletal Radiology Section, Klinikum Rechts Der Isar, Technical University of Munich, Ismaninger Strasse 22, 81675 Munich, Germany; 4 Kantonsspital Graubünden, KSGR, Loëstrasse 170, 7000 Chur, Switzerland

**Keywords:** Medical image segmentation, Deep learning, Multi-modal imaging, Unsupervised segmentation, Label variability

## Abstract

Medical image segmentation is challenging due to the need for expert annotations and the variability of these manually created labels. Previous methods tackling label variability focus on 2D segmentation and single modalities, but reliable 3D multi-modal approaches are necessary for clinical applications such as in oncology. In this paper, we propose a framework for generating reliable and unbiased labels with minimal radiologist input for supervised 3D segmentation, reducing radiologists’ efforts and variability in manual labeling. Our framework generates AI-assisted labels through a two-step process involving 3D multi-modal unsupervised segmentation based on feature clustering and semi-supervised refinement. These labels are then compared against traditional expert-generated labels in a downstream task consisting of 3D multi-modal bone tumor segmentation. Two 3D-Unet models are trained, one with manually created expert labels and the other with AI-assisted labels. Following this, a blind evaluation is performed on the segmentations of these two models to assess the reliability of training labels. The framework effectively generated accurate segmentation labels with minimal expert input, achieving state-of-the-art performance. The model trained with AI-assisted labels outperformed the baseline model in 61.67% of blind evaluations, indicating the enhancement of segmentation quality and demonstrating the potential of AI-assisted labeling to reduce radiologists’ workload and improve label reliability for 3D multi-modal bone tumor segmentation. The code is available at https://github.com/acurtovilalta/3D_LabelGeneration.

## Introduction

Image segmentation holds significant potential in medical imaging, enabling more precise diagnoses, treatment planning, and monitoring disease progression, therefore improving patient outcomes [1]. Medical image segmentation has a wide range of applications in healthcare. In clinical settings, it is essential to accurately delineate organs, tumors, and other anatomical structures. For example, segmentation of brain lesions aids in diagnosing and monitoring neurological diseases [2], while tumor segmentation is crucial for planning oncology treatments [3]. In addition, segmentation plays a key role in tracking disease progression over time, providing valuable insights for treatment evaluation and decision-making [4]. Beyond clinical applications, medical image segmentation is also instrumental in advancing medical research and enhancing the study of diseases.

Despite the advances in deep learning in image segmentation [5, 6], medical image segmentation remains challenging due to the lack of definitive gold standards for medical labels [7]. These labels, crucial for training supervised models, are often created manually and require domain experts with a high level of expertise, a resource that is both rare and difficult to access. Manual labeling is time-consuming [8, 9], and the resulting labels often vary significantly depending on the expert’s experience, leading to biased and subjective results [10, 11]. This variability complicates the creation of consistent ground truths, affecting the reliability and usefulness of segmentation models [10].

Previous work captured labeling behavior using a multivariate Gaussian distribution, integrating this with feature maps for probabilistic segmentation predictions [11]. Other methods used coupled convolutional neural networks to learn annotator reliability and the true segmentation label distributions from purely noisy observations [7]. However, these methodologies still rely on the details and quality of the annotations. Other recent strategies shifted towards unsupervised methodologies using reinforcement learning [12], self-ensembling [13], transfer learning [14–16], or foundation models [1, 17, 18] to enhance model generalization and perform segmentation tasks in unlabeled datasets. These methodologies predominantly utilize a single image modality and approach segmentation in a 2D manner.

Nevertheless, 3D models are crucial for clinicians, especially in oncology. These models provide detailed knowledge of tumor dimensions, morphology, and the extent of tissue involvement which is crucial for effective treatment and surgical planning [3]. Most research efforts in creating reliable 3D tumor models addressing label variability have focused on specific tumor entities such as the brain [19–21] or liver tumors [22], where the images exhibit rigid structures and the tumors occur in recurrent anatomical locations. These studies used unsupervised algorithms based on reconstruction to learn the anatomical structures and detect anomalies [23]. In contrast, bone tumor segmentation presents a greater challenge due to the heterogeneous nature of these tumors [24] and their occurrence in different anatomical sites, making it difficult for models to learn a consistent structural pattern.

This research aims to improve radiologists’ workflow in manual labeling and create unbiased labels for training any downstream task, which in our case is a 3D multi-modal bone tumor segmentation. This study investigates whether artificial intelligence can assist in label creation, therefore reducing radiologists’s efforts, improving label accuracy, and reducing inter- and intra-observer variability, ensuring reliable training of supervised segmentation, potentially achieving state-of-the-art results.

## Materials and Methods

The local institutional review and ethics board approved this retrospective study (N°48/20S), conducted in accordance with national and international guidelines. Informed consent was waived for this anonymized study. This article follows the Checklist for Artificial Intelligence in Medical Imaging (CLAIM) [25] guidelines.

The primary aim was to demonstrate the feasibility of generating reliable, unbiased labels assisted by artificial intelligence and radiologists. Secondarily, we tested our approach using multi-modal 3D MRIs for cartilaginous tumor segmentation aiming to reach state-of-the-art performance.

### Data

A dataset, from two different institutions, of 204 preoperative axial 3D MRIs from unique patients with cartilaginous tumors was utilized, including enchondromas, atypical cartilaginous tumors, and chondrosarcomas. Their morphological similarities make differentiation challenging [26, 27], justifying their inclusion in the same segmentation task. Malignant diagnoses were verified by histopathology as the standard of reference. Benign and intermediate lesions were either verified by histopathology or discussed in the local tumor board. Each case included T1- and T2-weighted MRIs. One expert (> 10 years of experience, J.N.) manually created 2D labels segmenting tumor tissue. Data processing involved dimension matching and z-normalization. The volumes were resized at 512 × 512 pixels with 32 depth slices. The dataset was split into training, validation, and testing sets in an 80/10/10 ratio. Patient overlap was avoided during data splitting to ensure unbiased results.

### AI-Assisted Label Computation Framework

#### Step 1: Unsupervised Multi-modal 3D Segmentation Model

A convolutional neural network based on differentiable feature clustering [28, 29] was adapted to 3D medical imaging [12]. The model was built from scratch, specifically tailored for multi-modal 3D segmentation. It consisted of three blocks of 3D convolutional layers, ReLU activation, and batch normalization. Two parallel CNNs, one for T1-weighted and another for T2-weighted MRI inputs, were employed without shared weights. Each CNN independently extracts modality-specific features, optimizing weights separately for each modality to preserve their unique characteristics. These features are then concatenated to create a unified embedding of 60 features per voxel before the clustering begins.

The clustering process followed an unsupervised learning strategy, combining feature similarity and spatial continuity. First, an argmax operator was applied to the feature embeddings produced by the CNN, forming a response map. Each voxel is assigned to the cluster corresponding to the embedded dimension with the highest response value, effectively grouping similar features into clusters. Feature similarity was then computed by the cross-entropy loss between the feature embeddings and the network’s response map measuring how well the network’s response map assigned feature embeddings with similar characteristics to the same cluster label. Spatial continuity promotes smoothness in cluster assignments by penalizing differences in cluster labels across neighboring pixels. It was computed using the L1-norm of the response map’s horizontal, vertical, and depth axes. The loss function combined feature similarity and spatial continuity [28, 29] balanced by a weight parameter μ set to 1. Initially, the network predicted 60 clusters, which were adaptively merged, reducing the number of clusters until convergence and allowing the model to generate a number of clusters that align with the content of the medical images.

The adaptation of the CNN and clustering framework to 3D data involved replacing traditional 2D convolutional layers with 3D convolutions, allowing the network to capture spatial dependencies across depth slices in addition to height and width. Similarly, batch normalization was extended to 3D, normalizing activations across channels for entire 3D patches rather than 2D slices. For the clustering process, the spatial continuity term was adapted to enforce smoothness across all three spatial dimensions: horizontal, vertical, and depth by incorporating the L1-norm of differences in cluster labels not only across neighboring pixels within slices but also between adjacent slices.

The training was conducted on 164 cases using PyTorch, with data augmentation (horizontal flips with 0.3 probability and affine rotations with a maximum of ± 15°) and early stopping to prevent overfitting. A batch size of 4 and a learning rate of 0.001 was used. The model was trained on one GPU (DGX Station A100, 80 GB; NVIDIA [30]) using stochastic gradient descent and Xavier initialization [28, 29].

#### Steps 2 and 3: Expert Supervision and AI-Assisted Label Generation

The unsupervised segmentation process generates multiple clusters within the image but is inherently unable to determine which clusters correspond to tumor tissue. To refine these segmentations, expert supervision is introduced to identify and select clusters associated with tumor tissue for refinement. As this study focuses on assessing the viability of the proposed methodology, implementing a fully interactive interface for expert-guided selection is beyond its current scope at this stage. Instead, this process was simulated using T1- and T2-weighted labels generated by experts. These labels are combined through element-wise addition and then multiplied with the unsupervised segmentation to isolate the tumor region. Random seeds are then strategically placed within the isolated region to mimic expert clicks on clusters. It is important to clarify that the label generation process does not strictly require manual tumor segmentation for its final output. In this step, manual labels are utilized solely to simulate the process of “clicking by an expert.”

**Fig. 1:**
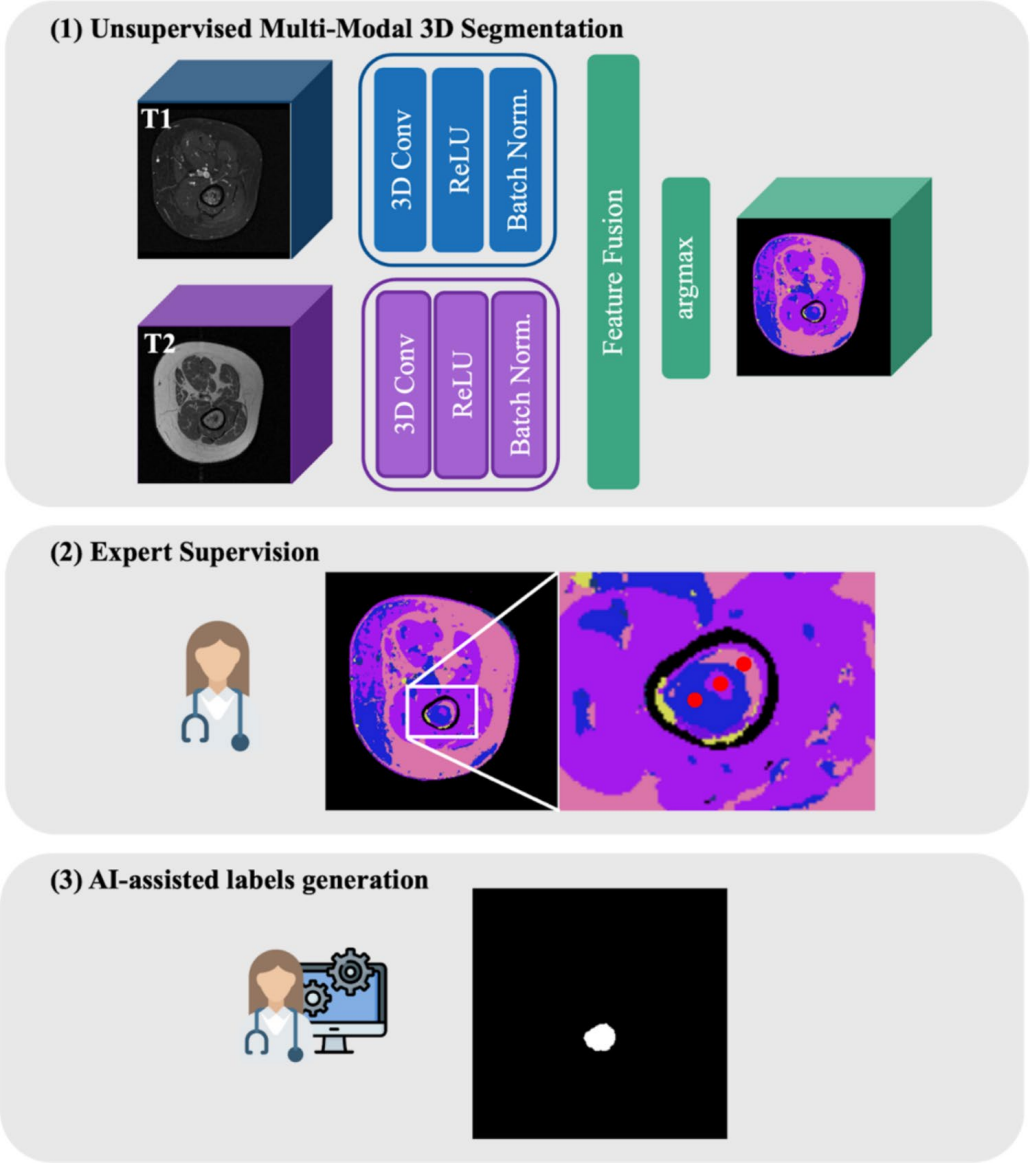
Deep learning framework workflow for AI-assisted segmentation label generation. Step (1) consists of a fully unsupervised segmentation model, followed by step (2) where the expert selects the clusters segmenting the tumor. The final label is generated in step (3) taking into account the input of the expert

A region-growing algorithm is applied using the seeds to expand within each cluster based on cluster labels rather than image intensity. This ensures that the process is driven by the inherent structure captured in the unsupervised segmentation. Separate masks are generated for each seed placed within the clusters. After the masks are generated, they are evaluated using a set of criteria designed to ensure accurate segmentation while minimizing over-segmentation. After testing various thresholds, the final criterion was established: only masks with a volume not exceeding three times the estimated tumor area were retained. This threshold was chosen to balance capturing the full extent of the tumor region while preventing the inclusion of excessive surrounding tissue. The final AI-assisted labels (AI-L) are derived by summing the valid masks (Fig. 1, steps (2) and (3)).

### Label Reliability Evaluation

To evaluate the reliability of AI-Ls in mitigating manual label variability, two identical supervised segmentation models were trained under the same conditions. The only difference was that one model was trained with expert labels (ELs), while the other was trained with AI-Ls. The predictions were then compared to evaluate the impact of the training labels on final segmentations.

#### Supervised Multi-modal 3D Segmentation Model

A 3D UNet [31] was adapted to integrate two MRI modalities by modifying the initial encoder with two input channels. Two models were trained in parallel, and the same data augmentation described previously was used, with a batch size of 4 and a learning rate of 0.001. Weight initialization is done by Xavier initialization with ADAM optimizer and trained on one GPU (DGX Station A100, 80 GB; NVIDIA [30]).

#### Blind Evaluation

To avoid bias in evaluating segmentations, direct comparison using ELs was discarded, opting instead for a blind evaluation. This involved three independent domain specialists—orthopedic surgeon (> 3 years of experience, B.S.), radiologist (> 10 years of experience, J.N.), and data scientist (> 4 years of experience, F.H.)—each evaluating 60 test cases. Segmentations from the two models were presented simultaneously in randomized order and colors, hiding their origins. To ensure consistency in evaluations, the experts first convened to establish a shared set of criteria for determining which segmentation was superior. The primary criterion was the completeness of tumor coverage, with a preference for segmentations that captured all tumor tissue, even if this resulted in some over-segmentation of healthy tissue. Secondly, they emphasized the importance of edge consistency, favoring segmentations with smooth and realistic delineation of tumor boundaries over those with irregular or jagged contours. Finally, the experts agreed that the segmentation should align with anatomical and radiological features, ensuring it was interpretable and clinically relevant. After establishing these criteria, experts selected the segmentation that represented better the bone tumor tissue delineation per each case, ensuring unbiased assessment.

## Results

### Dataset Characteristics

Table 1 presents the demographics, with an average age of 45.17 ± 15.68 years and 51.25% female representation. Enchondroma constitutes 49.51% of the cases, reflecting its commonality compared to rarer malignant tumors. The anatomical site of lesion distribution is presented, with most appearing in the extremities, indicating a varied and heterogeneous dataset. Additionally, the dataset exhibits heterogeneity in size and resolution, with T1-weighted MRIs ranging from 194 to 1024 pixels in side length and 11 to 52 slices, while T2-weighted MRIs ranging from 156 to 1680 pixels and 15 to 52 slices.

**Table 1:** Demographics of the dataset and anatomy site distribution. Age is presented as mean years ± SD, percentage of females, percentage distribution of cartilaginous tumor entity, and percentage distribution of anatomy

	Age		Female	Enchondroma	ACT	Chondrosarcoma		
	45.17 ± 15.68		51.25	49.51	22.55	27.94		
Thigh	Arm	Pelvis	Hand	Lower leg	Shoulder	Foot	Chest	Knee
27.5	22.5	13.33	10.83	7.92	7.5	5.83	2.5	2.09

### Downstream Task

#### Supervised Multi-modal 3D Bone Tumor Segmentation Model

Table 2 presents the performance metrics of the two identical supervised models on the test dataset, including the Dice similarity coefficient (DSC), false negative rate (FNR), and false positive rate (FPR), along with their corresponding standard deviations (SD). Each model was evaluated using the specific labels employed during training. The model trained with EL serves as a baseline, as it was developed using state-of-the-art methodologies.

**Table 2:** Supervised multi-modal 3D bone tumor segmentation test performance. Metrics: Dice similarity coefficient (DSC), false negative rate (FNR), and false positive rate (FPR)

	DSC (mean ± SD)	FNR (mean ± SD)	FPR (mean ± SD)
EL-trained model	0.47 ± 0.28	0.014 ± 0.01	0.0002 ± 0.01
AI-L-trained model	0.44 ± 0.23	0.016 ± 0.01	0.0003 ± 0.001

Despite relatively low DSC scores (0.47 ± 0.28 for EL-trained model and 0.44 ± 0.23 for AI-L-trained model), both models excelled in minimizing the FNR (0.014 ± 0.01 for EL-trained model and 0.016 ± 0.01 for AI-L-trained model), crucial for avoiding missed tumor tissue. The FPR was also low (0.0002 ± 0.01 for EL-trained model and 0.0003 ± 0.001 for AI-L-trained model), consistent with the background-heavy nature of the images.

**Fig. 2:**
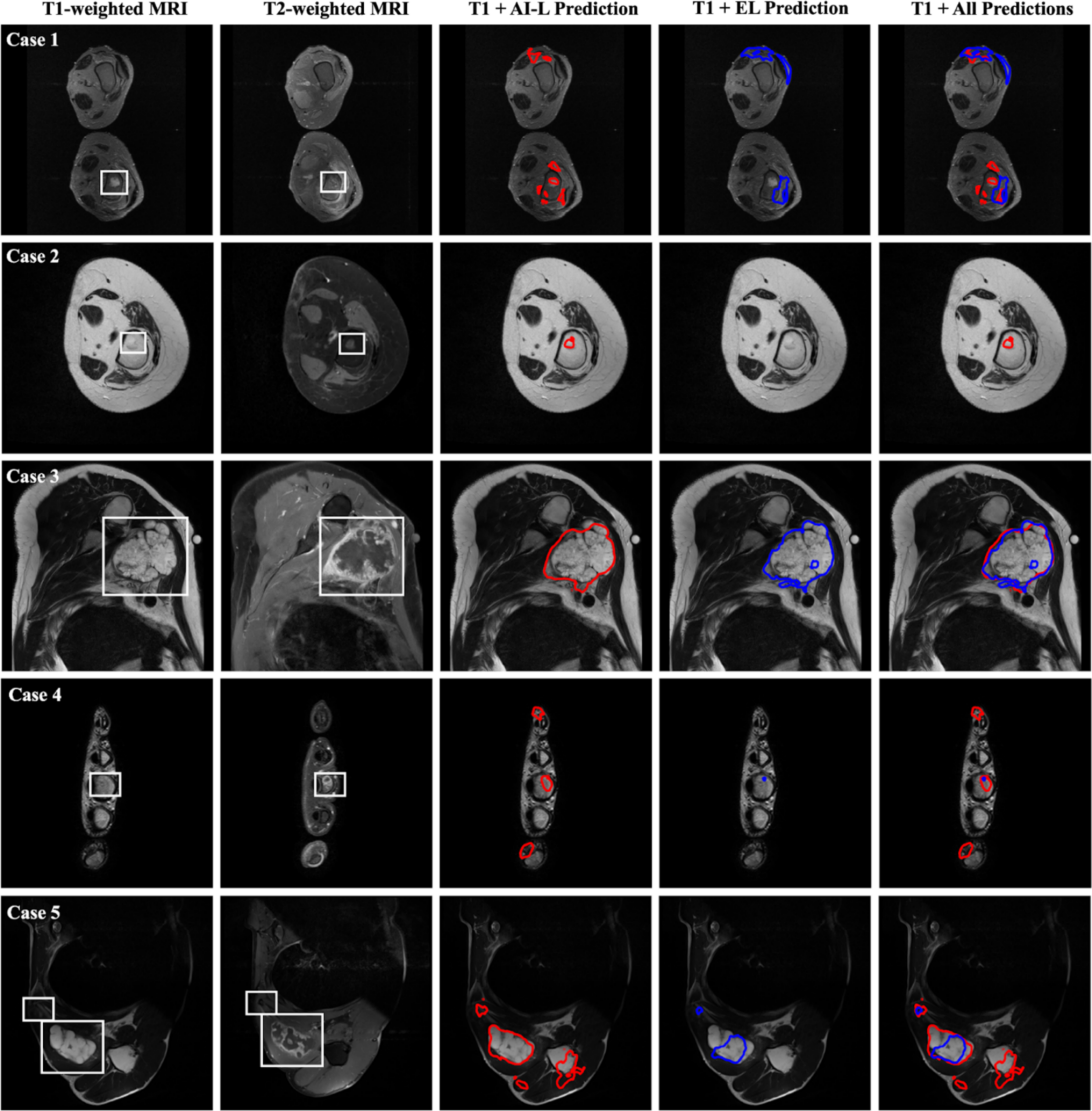
Supervised multi-modal 3D bone tumor segmentation model predictions. For each case, T1- and T2-weighted MRI images are presented as the inputs for the supervised models. The segmentation generated by the AI-L-trained model is shown in red, while the segmentation generated by the EL-trained model is shown in blue. The final image in each row overlays both segmentations on the T1 image for comparison

Additionally, Fig. 2 illustrates five different cases, each displaying the original T1- and T2-weighted MRI images, the segmentation predictions from the AI-L-trained model in red and the EL-trained model in blue. The final column shows an overlay of both predictions on the T1-weighted MRI for comparison. It is observed that the AI-L-trained model generally tends to over-segment (Case 1, 3, 4, 5), while the EL-trained model often under-segments (Case 1, 4, 5) or misses the tumor entirely (Case 2).

#### Blind Evaluation

Table 3 summarizes the outcomes of the blind evaluation conducted on 60 test segmentations. The table indicates the frequency with which each expert identified either the EL-trained model output or the AI-L-trained model output as the superior segmentation. The AI-L-trained model output was selected 109 out of 180 times (60.56%). Additionally, we calculated the majority vote for each case, requiring agreement from at least two evaluators. In this scenario, 37 out of 60 test cases (61.67%) had at least two experts agreeing that the AI-L-trained model provided the best segmentation. This evaluation demonstrated robustness, as complete agreement among experts was observed in 44 out of 60 cases (73.33%). Only in 16 out of 60 cases (26.67%), two experts differed from the third expert.

**Table 3:** Experts blind evaluation choices

	EL-trained model	AI-L-trained model
Expert 1	26	34
Expert 2	21	39
Expert 3	24	36
Total	71 (39.44%)	109 (60.56%)
Majority vote	23 (38.33%)	37 (61.67%)

### Evaluation of AI-Assisted Labels

#### Unsupervised Segmentation Assessment

Since the model is trained in an unsupervised manner, traditional performance metrics [32], which rely on actual labels, cannot be provided. Consequently, the performance of the unsupervised model in this context is best assessed visually.

**Fig. 3:**
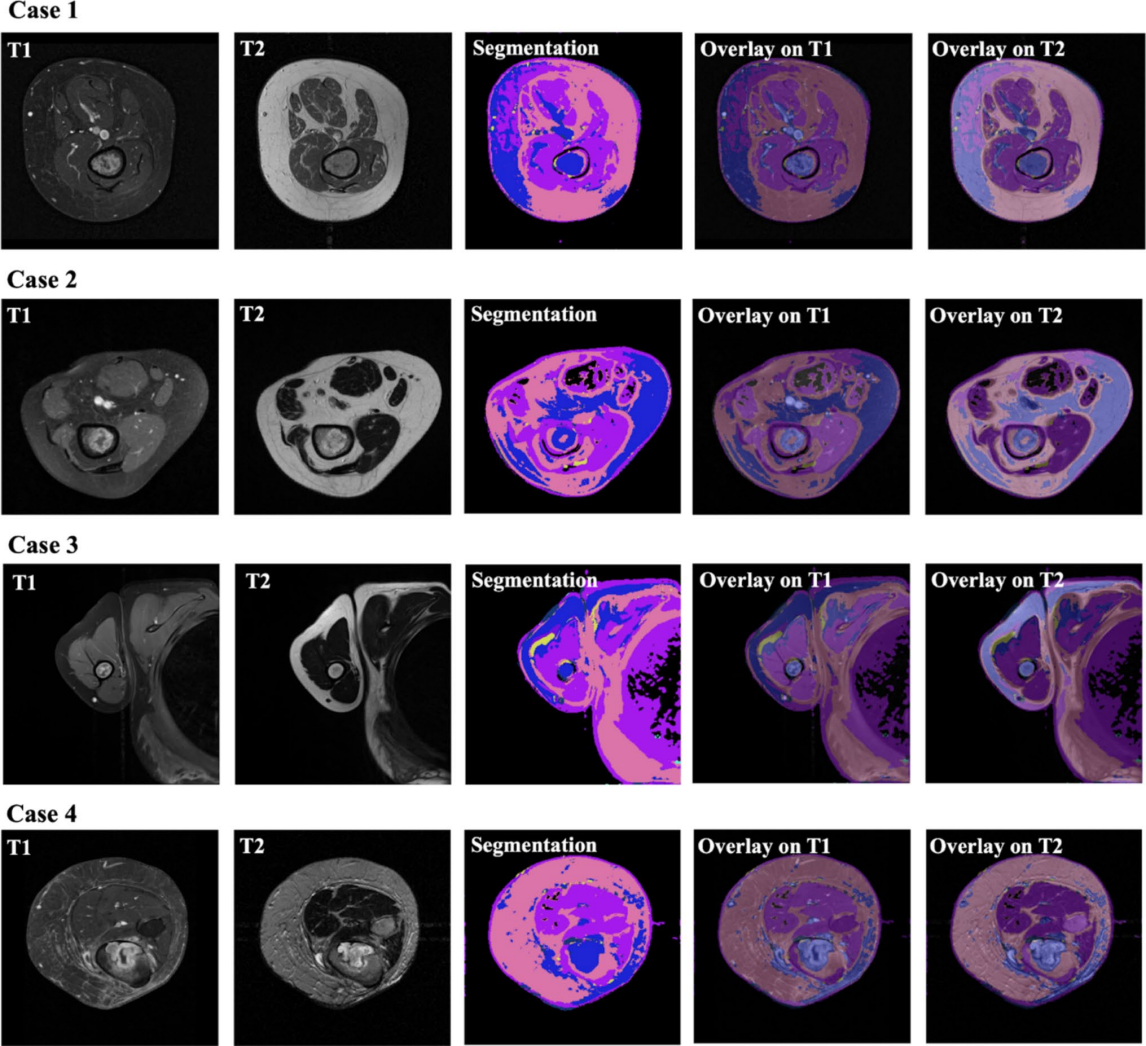
Unsupervised multi-modal 3D segmentation model output. Four different cases are depicted. For each case, T1- and T2-weighted MRI images are presented as the inputs of the model; then, the unsupervised segmentation is shown as well as its overlay on T1- and T2-weighted MRIs. Cluster colors are presented in Table 4

The model converged after 139 epochs, with early stopping triggered by a patience of 10 epochs. Upon reaching convergence, the final number of clusters was 7. Two of these clusters represented the background of the images and were therefore discarded for the analysis. The remaining five clusters are depicted in Table 4. These five clusters demonstrated consistent correspondence among slices and cases, despite some inaccuracies, as shown in Fig. 3. Specifically, cluster 1 consistently segmented tumor tissue across all cases, even though it appeared in other parts of the image as well. Cluster 2, although less frequently observed, also segmented tumor regions when it appeared. Cluster 3 predominantly segmented muscle tissue but also other tissues with the same signal intensities such as parts of the lung in Case 4 in Fig. 3. Cluster 4 appeared minimally in the segmentations, suggesting it does not play a major role in segmenting specific anatomy but might be attempting to identify for example vessel areas. Lastly, cluster 5 mainly segmented fat tissue.

**Table 4:**
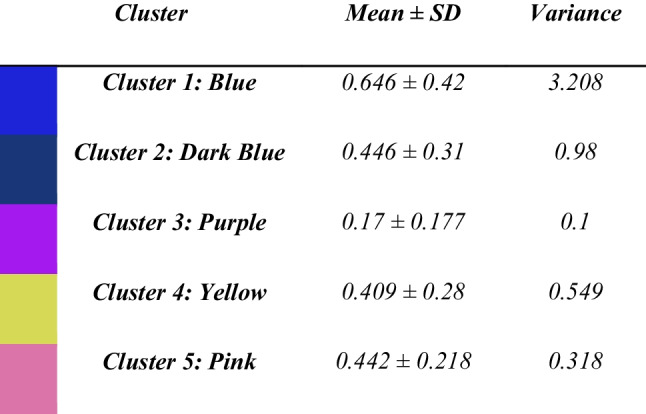
Intra-cluster homogeneity analysis in T1- and T2-weighted images across all volumetric cases

For quantitative evaluation, we conducted an intra-cluster homogeneity analysis (Table 4). This analysis assesses the consistency of pixel intensities within each cluster across the slices and cases, for both T1- and T2-weighted MRIs. Cluster 1, which corresponds to the tumor area, exhibited the highest variance among all clusters followed by Cluster 2 which also represents tumor regions. This finding aligns with clinical observations, as bone tumors are inherently heterogeneous [24]. This high variance in Cluster 1 and 2 underscores the model’s ability to capture the complex and variable nature of bone tumors, enhancing the reliability of the segmentations produced by the unsupervised model.

#### Expert-Supervised Refinement

**Fig. 4:**
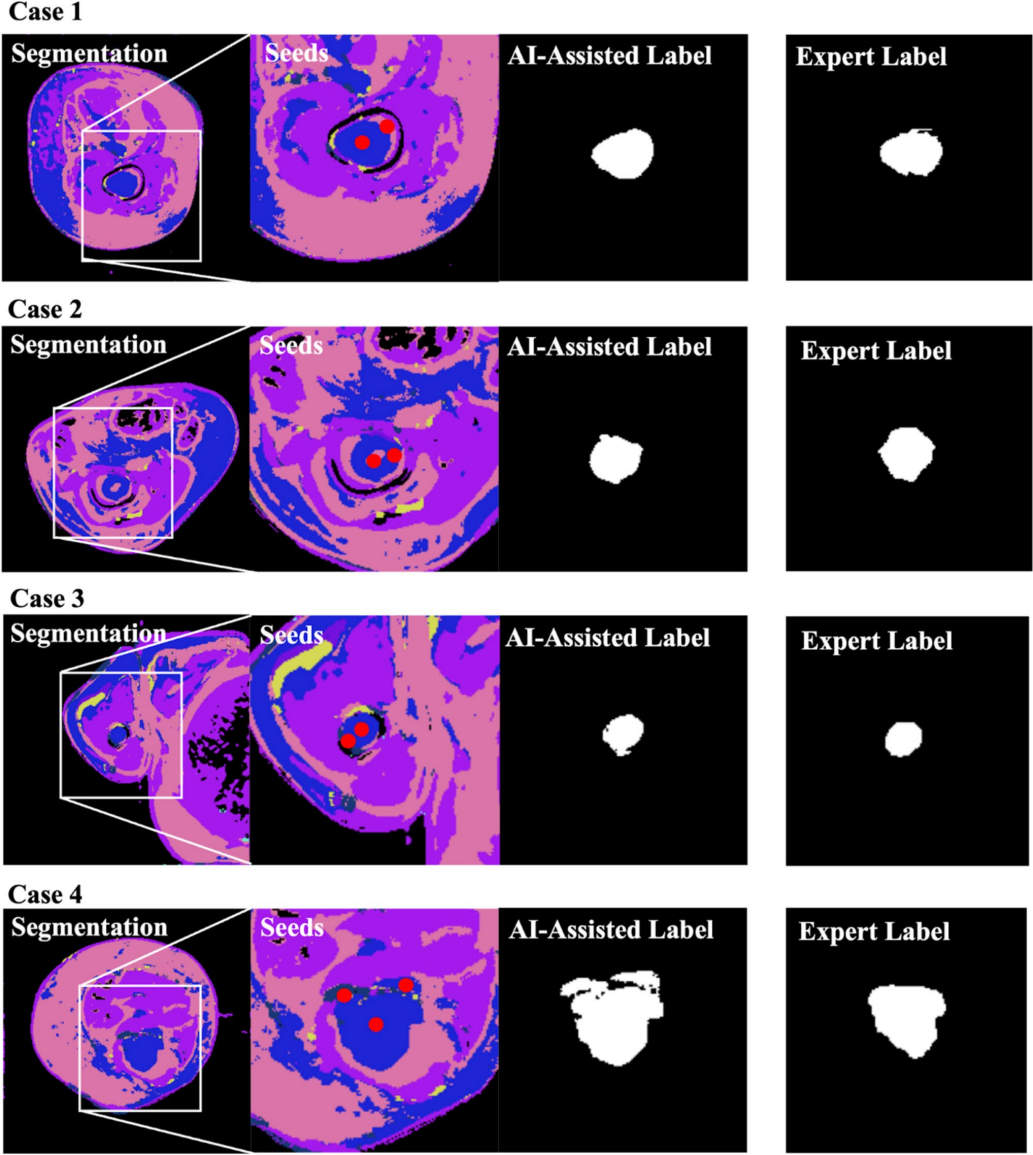
Refined AI-assisted labels with expert guidance. Four different cases are depicted, each showcasing the initial unsupervised segmentations, the expert-indicated seed points, and the final AI-assisted labels produced after refinement using these expert seeds

In Fig. 4, four cases are depicted with the resulting AI-L after expert refinement by setting seeds. For each case segmentation, seeds, AI-L, and EL are shown. Since AI-L are an improvement on ELs, overlap metrics highlight dissimilarities rather than similarities. The mean and SD overlap between the resulting AI-L and EL is 0.85 ± 0.15, indicating high similarity. However, the non-overlap areas might contribute to the AI-L improvement.

**Fig. 5:**
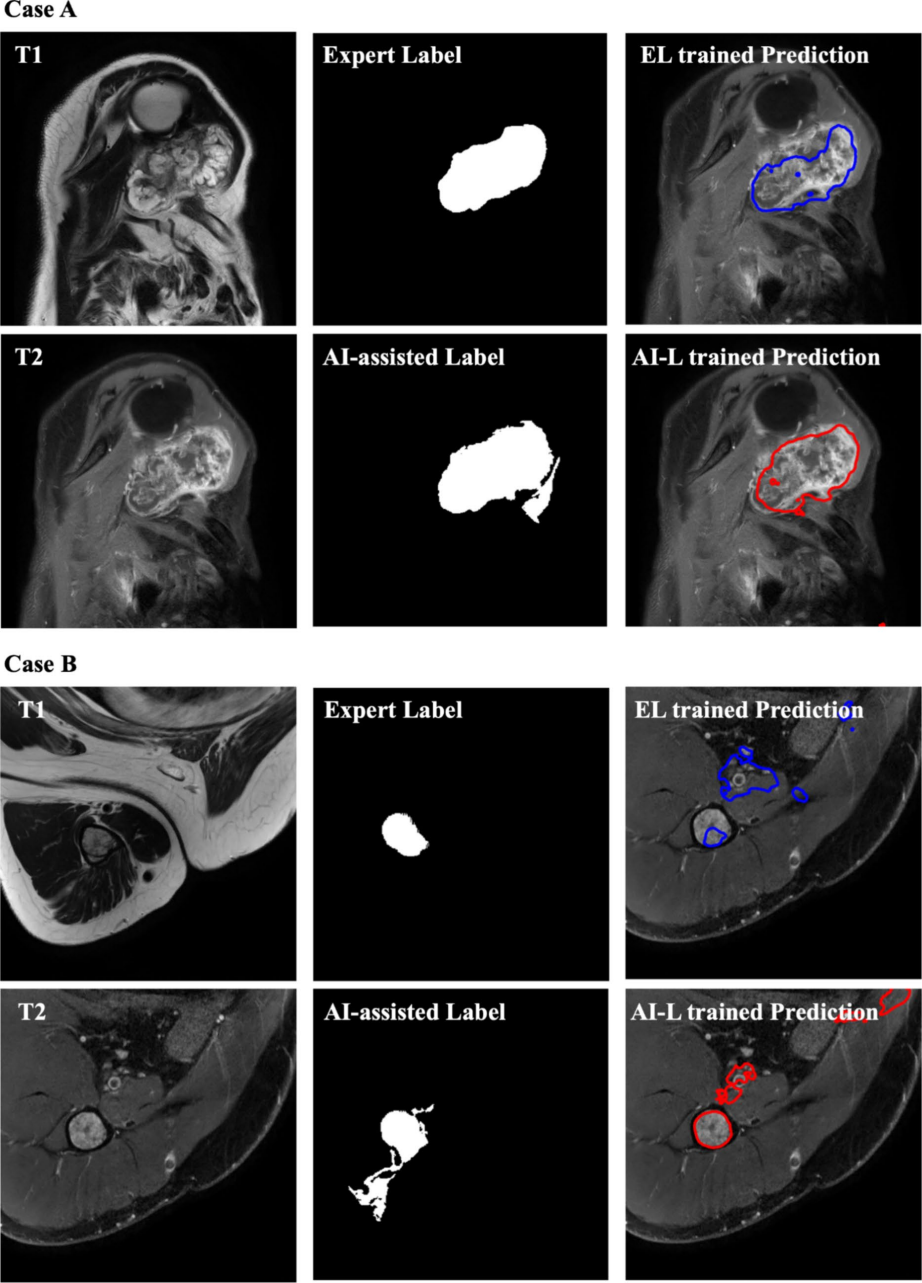
Comparison of AI-L and EL-trained model performance. This figure showcases two cases where the AI-L-trained model outperformed the EL-trained model based on a complete agreement between experts. For each case, the labels used for training are shown alongside the resulting predictions overlaid on the T2-weighted image

To further investigate the impact of these dissimilarities on the improvement of the AI-L-trained model, we analyzed 28 test cases where all three experts unanimously agreed on the superior performance of the AI-L-trained model. Even if these test cases were not used for training, they provide valuable insights into the model’s behavior. In the 28 cases, 21 (75%) had an overlap of at least 85%, while the remaining cases (7) had a mean and SD overlap of 0.70 ± 0.13, indicating differences between labels. In these instances, the AI-L model consistently over-segmented the tumor area, leading to improved results. Figure 5 presents two of these seven cases, illustrating the superior performance of the AI-L-trained model in accurately segmenting the regions of interest. In addition, Fig. 5 shows the tendency of AI-L to over-segment the tumor area compared to the EL.

## Discussion

Our framework successfully generated semi-supervised labels with minimal radiologist inputs. These labels effectively trained a segmentation model achieving state-of-the-art performance. Notably, in the blind evaluation, 61.67% of the test cases had at least two experts agreeing that the AI-L-trained model provided the superior segmentation, suggesting that AI-assisted labels provide additional information that enhances model performance. Despite the superior performance of the AI-L model, both the AI-L and baseline models (EL model) occasionally misidentified the tumor site and incorrectly labeled other areas, such as normal bone or vessels, as pathological. This indicates that the AI-L model is meeting state-of-the-art performance, as the baseline model exhibited similar errors.

To understand the improvements brought by AI-L, we started by analyzing the unsupervised segmentations. Initially, the unsupervised model segmented 60 clusters, which were reduced systematically to 7 clusters, aligning with expected tissue types: background, bone, muscle, fat, tumor, cartilage, and blood vessels. Tumors were consistently detected mainly in the blue cluster and minimally in the dark blue cluster, both of which showed high pixel intensity variance, a clinically aligned finding. However, these segmentations were not entirely accurate, sometimes misclassifying non-tumor regions as tumors, highlighting the need for expert refinement.

In our framework, radiologists refine these segmentations by identifying clusters containing tumor areas, significantly reducing their effort compared to manual labeling. We simulated this process using expert-provided labels, resulting in a high overlap in training labels, indicating that our framework is able to create accurate segmentation labels.

Finally, we conducted an in-depth analysis of cases where experts unanimously preferred the AI-L-trained model. In these cases, we compared the AI-L and the EL that would have been used for training. In 75% of the cases, an overlap of at least 85% (21/28) was observed. In the remaining 25% (7/28), the mean overlap was 0.70 ± 0.13. The dissimilar cases revealed that AI-L over-segmented the tumor, including other parts of the image. This over-segmentation might act as positive noise, potentially beneficial in tasks with limited data. This finding suggests that over-segmentation is preferable to under-segmentation. Therefore, in cases of uncertainty, when radiologists perform manual labeling, over-segmentation would be preferable.

In contrast to related studies focusing on label reliability in 2D approaches [12, 13] using a single image modality, our work focuses on 3D segmentation using multi-modal MRI to enhance the imaging information for tumor assessment, particularly bone tumors. This approach aligns with the diagnostic processes in this field, which often involve the integration of multi-modal imaging data to achieve a detailed evaluation [3, 33, 34].

The proposed methodology is adaptable to various volumetric datasets, requiring two corresponding modalities. Implementing this method demands substantial computational resources, especially for 3D data, due to the size and complexity of volumetric data processing. Thus, careful evaluation and allocation of computational resources are essential when applying this methodology to volumetric datasets. Despite the adaptability of the proposed methodology, a limitation of this study is the absence of formal external validation on an independent dataset. While this is an important step for robust evaluation, we believe the heterogeneity of our dataset partially addresses generalizability concerns. Our dataset includes cases from two distinct institutions with varying imaging protocols, equipment, patient populations, and multiple anatomical sites rather than being restricted to a single region or imaging context. This diversity introduces variability similar to that of external datasets, demonstrating the framework’s potential robustness across different clinical and imaging environments.

This study encountered several limitations, primarily due to the inherently challenging task of bone tumor segmentation, which is difficult even for experts to precisely define tumor boundaries. Additionally, the limited dataset size poses risks of bias and affects the generalizability of the results, restricting definitive conclusions about model performance. The absence of pre-trained volumetric weights resulted in slow training times, as training from scratch is computationally intensive. Pre-trained weights could mitigate the impact of small datasets and accelerate training.

Another significant limitation is the complexity of visually evaluating unsupervised and semi-supervised methodologies, which introduces subjectivity and variability. Next steps should prioritize developing more objective evaluation strategies to improve reliability and consistency in segmentation assessment. One potential approach is to generate high-resolution ground-truth annotations created by multiple experts, ensuring a robust reference standard. These annotations could then be aggregated using techniques such as a majority pixel-wise vote to reduce individual biases. Quantitative segmentation metrics could then be applied to compare the model outputs against this aggregated ground truth, providing a more standardized and reproducible evaluation framework.

Despite these challenges, our framework potentially reduces radiologists’ workload by requiring only minimal expert involvement in the form of seed placement within tumor regions. The approach significantly reduces the workload for radiologists compared to full manual segmentation. The precision is maintained because the selection of clusters is guided by expert input rather than being determined solely by the model, ensuring that the segmentation process aligns with clinical expertise.

Future efforts should focus on enhancing the unsupervised segmentation model by exploring improvements in architecture, optimization techniques, and loss functions to achieve more accurate segmentation. Specifically, the model should be refined to distinctly segment bone and cartilaginous tumors, as current results do not show a clear differentiation between these tissues. Additionally, future development aims to eliminate the use of manual labels by creating an interface where experts can set seed points directly. Additionally, integrating reinforcement learning to improve label generation is a promising advancement. In this approach, experts’ seed points could serve as rewards and incorrect areas as punishments, enabling a neural network to learn from this feedback and eventually set seeds autonomously.

### Conclusion

In conclusion, our framework efficiently generated labels with minimal radiologist input, training a segmentation model that achieved state-of-the-art performance. The AI-L-trained model outperformed the EL-trained model, indicating enhanced performance due to AI-assisted labels. Qualitative analysis showed that tumors were primarily detected in clinically plausible clusters showcasing the reliability of AI-L, though expert refinement was necessary to correct misclassifications. Despite challenges such as limited dataset size and subjective evaluations, our approach reduces radiologists’ workload and has the potential for broader application.

## Data Availability

The data used in this study are not publicly available due to institutional and patient confidentiality regulations. Access may be granted under specific conditions. Researchers interested in accessing the data may contact the corresponding author for further inquiries.

## Abbreviations

AI-Ls: Artificial intelligence–assisted labels
ELs: Expert labels
DSC: Dice similarity coefficient
SD: Standard deviation
FNR: False negative rate
FPR: False positive rate
